# Infection Risk for Persons Exposed to Highly Pathogenic Avian Influenza A H5 Virus–Infected Birds, United States, December 2014–March 2015

**DOI:** 10.3201/eid2112.150904

**Published:** 2015-12

**Authors:** Carmen S. Arriola, Deborah I. Nelson, Thomas J. Deliberto, Lenee Blanton, Krista Kniss, Min Z. Levine, Susan C. Trock, Lyn Finelli, Michael A. Jhung, the H5 Investigation Group

**Affiliations:** Centers for Disease Control and Prevention, Atlanta, Georgia, USA (C.S. Arriola, L. Blanton, K. Kniss, M.Z. Levine, S.C. Trock, L. Finelli, M.A. Jhung);; United States Department of Agriculture, Washington, DC, USA (D.I. Nelson);; United States Department of Agriculture, Fort Collins, Colorado, USA (T.J. Deliberto)

**Keywords:** avian influenza, influenza, avian influenza virus, viruses, highly pathogenic avian influenza virus, human exposure, risk of influenza illness, H5 subtype, birds, United States

## Abstract

Newly emerged highly pathogenic avian influenza (HPAI) A H5 viruses have caused outbreaks among birds in the United States. These viruses differ genetically from HPAI H5 viruses that previously caused human illness, most notably in Asia and Africa. To assess the risk for animal-to-human HPAI H5 virus transmission in the United States, we determined the number of persons with self-reported exposure to infected birds, the number with an acute respiratory infection (ARI) during a 10-day postexposure period, and the number with ARI who tested positive for influenza by real-time reverse transcription PCR or serologic testing for each outbreak during December 15, 2014–March 31, 2015. During 60 outbreaks in 13 states, a total of 164 persons were exposed to infected birds. ARI developed in 5 of these persons within 10 days of exposure. H5 influenza virus infection was not identified in any persons with ARI, suggesting a low risk for animal-to-human HPAI H5 virus transmission.

## Abstract 2

No infections have been reported among >100 exposed persons, suggesting a low risk for animal-to-human transmission.

Poultry infections with highly pathogenic avian influenza (HPAI) A H5 viruses have rarely been reported in the United States and have previously occurred as localized events (*1*,*2*). However, during December 15, 2014–March 31, 2015, a total of 60 HPAI H5 outbreaks in wild, captive, and domestic birds were identified in 13 states (*3*).

HPAI H5 viruses emerge sporadically in poultry as a result of interspecies transmission from wild to domestic birds (*4*,*5*). HPAI H5 viruses have caused thousands of outbreaks in poultry worldwide (*6*). Avian influenza viruses have evolved to bind to receptors in birds that differ from those in humans (*7*); therefore, the ability of avian influenza viruses to infect humans is limited. Nonetheless, human infections with avian influenza viruses have occurred. Most human infections have been caused by HPAI subtype H5N1 viruses in several countries and by low pathogenic avian influenza (LPAI) subtype H7N9 virus, primarily in China (*8*,*9*). Often, human infection with avian influenza virus results in severe disease (*10*). For instance, the H5N1 virus found in Asia (referred to here as Eurasian lineage H5N1) has been documented to cause severe disease in humans (*11*). Human infection with Eurasian lineage H5N1 virus was first documented in 1997 in poultry workers in Hong Kong, where the most frequent exposures were touching poultry or poultry parts and butchering poultry (*12*,*13*). In that outbreak, indirect exposures (e.g., feeding poultry, cleaning poultry stalls) were also associated with the presence of H5N1 antibodies in humans (*12*). To date, Eurasian lineage H5N1 virus has caused >800 human infections in Africa, Asia, and Europe, resulting in a 60% case fatality rate. Most of the H5N1 case-patients reported exposure to infected poultry at live bird markets or backyard farms (*14*). The first human cases of LPAI H7N9 virus infection were documented in China in 2013 (*8*); since then, >600 human infections and a case fatality rate of ≈36% have been reported. Like human HPAI H5N1 virus infections, human infections with LPAI H7N9 have been associated with exposure to infected poultry (*8*,*9*).

Of the 3 HPAI H5 subtype viruses recently identified in the United States (H5N1, H5N2, and H5N8) (*15*), only H5N8 virus has been identified previously in birds in Europe and Asia (*16*). One hypothesis for the recent emergence of these viruses in US birds attributes the arrival of H5N8 virus to migratory birds coming from Russia by the Pacific flyway (*16*,*17*). Once in North America, this H5N8 virus purportedly mixed with circulating North American LPAI viruses to generate 2 new reassortant HPAI viruses: subtype H5N2 and H5N1 viruses. In both of these new viruses, the hemagglutinin component originated from the migrant H5N8 virus, and the neuraminidase component originated from circulating LPAI viruses (*18*). Therefore, these 2 new viruses are genetically different from HPAI viruses identified in Asia. Of note, these new H5 viruses found in North American birds so far have not been associated with human infections. To help assess the risk of transmission of these viruses from birds to humans, we describe human exposure to HPAI H5 virus–infected birds during December 2014–March 2015 in the United States. This investigation was conducted as part of a public health response; thus, in accordance with federal human subjects protection regulations, it was not considered to be human subjects research.

## Methods

We identified HPAI H5 virus detections (i.e., laboratory-confirmed infections) in US birds by using reports made to the Animal and Plant Health Inspection Service, United States Department of Agriculture (USDA); the US Geological Survey, United States Department of Interior (DOI); the US Fish and Wildlife Service, DOI; and the National Flyway Council. These reports included the following information: county, state, confirmation date, influenza virus subtype, species and quantity of affected birds, and setting (wild, captive wild, backyard poultry, or commercial poultry). For the purpose of this investigation, we grouped detections of HPAI H5 virus–infected birds into outbreaks. We considered all detections in wild birds from specimens collected on the same day and in the same county to be 1 outbreak; those occurring on different dates or in different counties were classified as separate outbreaks. We considered multiple detections in captive wild birds and domestic flocks to be a single outbreak if the same HPAI H5 virus was detected in birds housed at the same location within 5 days of a prior detection.

For each outbreak reported, we contacted state and local public health departments to request information regarding human exposures. This information consisted of the number of persons who reported being exposed to possibly infected birds and the number of persons in whom acute respiratory infection (ARI) or other signs or symptoms compatible with avian influenza developed during a 10-day postexposure monitoring period. ARI was defined as >2 signs or symptoms of respiratory infection (i.e., fever, cough, runny nose or nasal congestion, sore throat, or difficulty breathing). Signs and symptoms considered compatible with avian influenza were eye tearing, irritation or redness, fatigue, muscle or body aches, headache, nausea, vomiting, diarrhea, stomach pain, and joint pain. Monitoring was conducted by state or local health department via direct observation or telephone call. We also requested a narrative description of the nature of exposure to potentially infected birds, when available. Hunters in affected areas were asked to submit their harvested birds for anonymous testing for HPAI H5 virus infection at USDA’s National Veterinary Services Laboratories. Because the testing was anonymous, the number of persons exposed to each hunter-harvested bird was largely not available to state and local public health departments; thus, we assumed that 1 person was exposed to an H5 virus-infected bird for each bird identified.

For persons in whom ARI developed during the monitoring period, we asked health departments to collect respiratory specimens for real-time reverse transcription PCR (rRT-PCR) testing at state public health laboratories. Specimens were obtained by using a nasopharyngeal swab or a nasal aspirate or wash or the combination of a nasal or nasopharyngeal swab with an oropharyngeal swab. If respiratory specimens were unavailable within 7 days of illness onset, we evaluated patients with ARI by performing serologic testing at the Centers for Disease Control and Prevention (CDC; Atlanta, GA, USA).

For rRT-PCR testing, specimens were first screened for universal detection of type A and B influenza viruses and human RNase P gene (InfA, InfB and RP primers, respectively), according to CDC protocol described elsewhere (*19*). Influenza A–positive specimens were tested by using the following primers: H1, H3, pdmInfA (2009 pandemic influenza A), pdmH1 (2009 pandemic H1), and H5. Paired serum samples were tested by microneutralization and hemagglutination inhibition assays, using horse erythrocytes, according to international standards (*20*). The following 2 viruses were used in both assays: A/np/WA/40964/2014 (an H5N2 virus isolated from the index case in birds) and A/gyrfalcon/WA/41088–6/2014 (an H5N8 virus isolated from the index case in birds). The following 3 viruses were used in the microneutralization assay only: A/California/07/2009 (an H1N1 vaccine strain and circulating strain), A/TX/50/2012(H3N2) (an H3N2 vaccine strain), and A/SW9715293/2013 (represents a currently circulating H3N2 strain).

We received information from USDA regarding exposures of persons involved in flock depopulation efforts. We considered these exposures separately because persons involved in depopulation efforts were recommended to wear personal protective equipment (PPE) to decrease the risk of transmission (*21*).

## Results

During December 15, 2014–March 31, 2015, a total of 60 outbreaks of HPAI H5 outbreaks were investigated by the Animal and Plant Health Inspection Service, USDA; the National Wildlife Health Center, US Geological Survey, DOI; state agriculture departments; or state natural resources departments. The outbreaks were caused by H5N2 virus (n = 37), H5N8 virus (n = 22), and H5N1 (n = 2) (these numbers total 61, not 60, because 3 outbreaks had a combination of viruses [1 H5N1/H5N2 and 2 H5N2/H5N8] and viruses in 2 outbreaks were not subtyped, but specimens were diagnosed as H5). Of the 60 outbreaks, 38 (63%) occurred in wild birds, 9 (15%) in backyard flocks, 8 (13%) in commercial flocks, and 5 (8%) in captive wild birds (Table). A total of 41 counties in 13 states reported HPAI infections in birds (Figure).

**Table:** Location and characteristics of highly pathogenic avian influenza A H5 virus outbreaks among birds and minimum number of exposed persons, United States, December 15, 2014–March 31, 2015

Variable	No. (%) outbreaks among birds, n = 60	No. (%) virus-exposed persons,* n = 164
State		
Arizona	1 (2)	2 (1)
California	8 (13)	30 (18)
Idaho	8 (13)	16 (10)
Kansas	2 (3)	5 (3)
Minnesota	3 (5)	17 (10)
Missouri	4 (7)	26 (16)
Montana	1 (2)	2 (1)
New Mexico	1 (2)	1 (1)
Nevada	1 (2)	5 (3)
Oregon	10 (17)	20 (12)
Utah	1 (2)	1 (1)
Washington	19 (32)	37 (23)
Wyoming	1 (2)	2 (1)
Influenza virus subtype†		
H5N1	2 (3)	3 (2)
H5N2	37 (59)	103 (63)
H5N8	22 (35)	56 (34)
H5‡	2 (3)	2 (1)
Outbreak setting		
Wild	38 (63)	64 (39)
Captive	5 (8)	13 (8)
Backyard farm	9 (15)	25 (15)
Commercial farm	8 (13)	62 (38)
No. birds per outbreak		
1–5	42 (70)	71 (43)
6–500	9 (15)	29 (18)
>500	9 (15)	64 (39)

*Excludes persons who participated in culling activities. †Three outbreaks involved a combination of influenza virus subtypes: H5N1/H5N2 (n = 1) and H5N2/H5N8 (n = 2). ‡No virus was isolated, but specimens were positive by the H5 (intercellular adhesion gene cluster) PCR assay, which targets the Eurasian H5 clade 2.3.4.4 viruses that were detected in the United States in December 2014.

**Figure:**
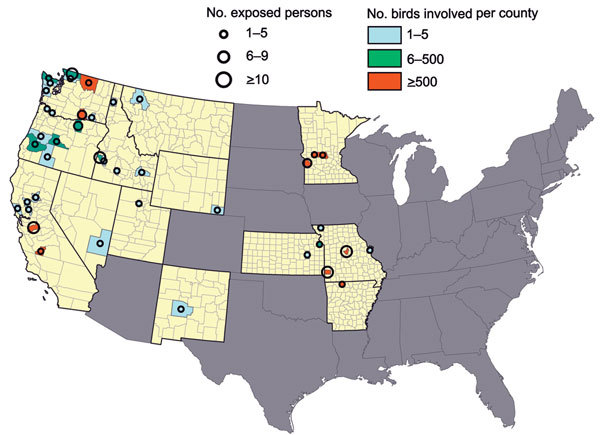
Number of highly pathogenic avian influenza A H5 virus–infected birds and minimum number of exposed persons by state and county, United States, December 15, 2014–March 31, 2015. Yellow indicates states in which outbreaks occurred.

We identified 164 human exposures: 103 (63%) were associated with H5N2 virus, 56 (34%) with H5N8 virus, 3 (2%) with H5N1 virus, and 2 (1%) with H5. Of the 164 exposed persons, 13 (8%) were exposed to captive wild birds, 25 (15%) were exposed to poultry in backyard farms, 62 (38%) were exposed to poultry in commercial flocks, and 64 (39%) were exposed to wild birds (Table).

We received information describing exposures for 60 of the 164 persons; 44 (73%) had exposure to infected birds while not wearing PPE (e.g., while removing dead birds, collecting eggs, cleaning coops, or feeding birds), and 16 (27%) had exposure while wearing recommended PPE or had unclear exposures. ARI developed in 5 (3%) of the 164 exposed persons within 10 days of their last contact with infected birds; 4 of the 5 tested negative for influenza virus by rRT-PCR. The remaining patient with ARI had paired serum samples collected 7 days and 21–28 days after exposure, respectively. This person had serologic evidence of seasonal influenza A(H3N2) virus infection, but had no serologic evidence of infection with an HPAI H5 virus.

An additional 29 persons were involved with depopulation activities of affected flocks while wearing recommended PPE. None of those persons reported ARI within 10 days after last exposure.

## Discussion

Within 4 months of the first outbreak of HPAI H5 viruses among birds in the United States, we identified >100 potential human exposures to infected birds. We found no evidence of human infection with these viruses among exposed persons. Our findings suggest that transmission of these HPAI viruses from birds to humans in exposure settings similar to those in this report may be uncommon.

Previous studies have shown that transmission of other H5 viruses from infected birds to humans has rarely occurred in Europe, Asia, and Africa (*10*,*22*,*23*). Exposures in those studies also likely occurred in a different context in many circumstances and may have had higher transmission likelihood than the exposures described in this report. For instance, a study in Egypt found that 12 (86%) of 14 households with an H5N1 virus–infected member lacked appropriate disposal of slaughtered poultry waste (e.g., feathers, viscera), and only 1 of 56 households reported using disinfectants when cleaning poultry-contaminated surfaces (*24*). In addition, poultry exposure in these areas frequently involved unprotected and prolonged contact with unconfined poultry in poor sanitary conditions, situations that are infrequently found in the United States (*24*).

We acknowledge the following limitations of this study. First, the number of persons determined to be exposed in this investigation is likely an underestimate because we did not have complete information on human exposures for all outbreaks. Specifically, hunter-harvested birds were in many cases reported anonymously, and our assumption of 1 exposed person per bird may be incorrect. Second, no systematic testing was performed for exposed persons in whom ARI did not develop, and it is possible that we failed to identify instances of bird-to-human HPAI H5 virus transmission that resulted in subclinical infection. However exposed persons were monitored carefully for illness, and previously reported human infections with related HPAI H5 viruses have resulted in severe and prominent symptoms (*25*). Third, we were unable to collect detailed exposure information for all exposed persons; thus, we could not describe the precise nature or duration of exposures we report. Fourth, there have been relatively few HPAI H5 virus exposure events in the United States to date, which limits our ability to provide a reliable quantitative estimate of the zoonotic risk posed by these viruses. Last, because some reporting is delayed, additional outbreaks of H5 in wild birds may be identified retrospectively within the timeframe of this investigation; additional H5 outbreaks in all bird categories will also likely continue after March 31, 2015.

Although this early assessment suggests that the risk of bird-to-human transmission of HPAI H5 viruses in the United States may be low, the CDC recommends vigilance when considering future human exposures to birds that are or may be infected. Similar HPAI H5 viruses, such as Eurasian H5N1 and H5N6 viruses, have caused severe illness and death in humans in Europe, Asia, and Africa (*13*,*26*), and these newly identified US HPAI viruses should be regarded as having the potential to cause severe disease in humans until shown otherwise. The best way to prevent human infection with avian influenza A viruses is to avoid unprotected contact with sick or dead infected poultry. Persons who have been exposed to HPAI-infected birds should be monitored for 10 days after last exposure and be tested for influenza as soon as possible after illness onset if respiratory symptoms develop. Exposed persons may also be offered influenza antiviral chemoprophylaxis. Additional guidance on testing, monitoring, and chemoprophylaxis is available at http://www.cdc.gov/flu/avianflu/guidance-exposed-persons.htm.

HPAI H5 virus outbreaks in US birds will likely continue, and additional reassortment with North American viruses may also occur. Although the risk of virus transmission to humans appears to be low, each exposure incident should be reported immediately and investigated collaboratively by animal and human health partners. A rapid response to any potential human cases of HPAI H5 infection in the United States is critical to prevent further cases, evaluate clinical illness, and assess the ability of these viruses to spread among humans.
